# Disrupting the Gut–Brain Axis: How Artificial Sweeteners Rewire Microbiota and Reward Pathways

**DOI:** 10.3390/ijms262010220

**Published:** 2025-10-21

**Authors:** Roberto Coccurello

**Affiliations:** 1 Institute for Complex Systems (ISC), National Research Council (C.N.R.), 00185 Rome, Italy; roberto.coccurello@cnr.it; 2 European Center for Brain Research—Institute for Research and Health Care (IRCCS) Santa Lucia Foundation, 00143 Rome, Italy

**Keywords:** artificial sweeteners, non-caloric sweeteners, gut–brain axis, microbiota dysbiosis, hedonic feeding, dopamine signaling, oxytocin, hypothalamus, ventral tegmental area, reward

## Abstract

Artificial sweeteners, or non-caloric sweeteners (NCSs), are widely consumed as sugar substitutes to reduce energy intake and manage obesity. Once considered inert, accumulating evidence now shows that NCSs interact with host physiology, altering gut microbiota composition and neural circuits that regulate feeding. This review synthesizes current knowledge on how NCSs disrupt the gut–brain axis (GBA), with particular focus on microbiota-mediated effects and neural reward processing. In homeostatic regulation, NCS-induced dysbiosis reduces beneficial taxa such as *Akkermansia muciniphila* and *Faecalibacterium prausnitzii*, diminishes short-chain fatty acid production, impairs gut barrier integrity, and promotes systemic inflammation. These changes blunt satiety signaling and favor appetite-promoting pathways. Beyond homeostasis, NCSs also rewire hedonic circuits: unlike caloric sugars, which couple sweet taste with caloric reinforcement to robustly activate dopaminergic and hypothalamic pathways, NCSs provide sensory sweetness without energy, weakening reward prediction error signaling and altering neuropeptidergic modulation by orexin, neurotensin, and oxytocin. Microbial disruption further exacerbates dopaminergic instability by reducing precursors and metabolites critical for reward regulation. Together, these top-down (neural) and bottom-up (microbial) mechanisms converge to foster maladaptive food seeking, metabolic dysregulation, and increased vulnerability to overeating. Identifying whether microbiome-targeted interventions can counteract these effects is a key research priority for mitigating the impact of NCSs on human health.

## 1. Introduction

### 1.1. Artificial Sweeteners and Public Health

Artificial sweeteners, also known as non-nutritive or non-caloric sweeteners (NCSs), provide high-intensity sweetness with minimal or no caloric contribution and can contribute to reduced overall energy intake when used as substitutes for sugar, although the magnitude of this effect varies across studies. Commonly used NCSs include sucralose, aspartame, saccharin, acesulfame potassium (Ace-k), and natural alternatives like steviol glycosides [1]. They were considered inert compounds with no systemic effects and were thought to offer a safe and effective strategy to manage obesity and related metabolic disorders. Their growing worldwide consumption and, in particular, their extensive incorporation into food and beverages has grown in parallel with global public health initiatives aimed at reducing added sugar consumption with the purpose of curbing obesity (e.g., “globesity”) and limiting the rising diabetes rates [1,2,3]. Their widespread use reflects this perception of safety. With increasing interest of consumers in “sugar-free” and “diet” foods and products, NCSs have become integral components of modern dietary patterns across diverse populations and age groups, especially for the dietary characteristics of the Westernized diet (WD) [4,5].

### 1.2. From Safety to Complexity

In recent years, however, growing evidence has challenged the idea that NCSs are metabolically neutral. Despite regulatory assurances of safety and their classification as Generally Recognized as Safe (GRAS) by agencies such as the U.S. Food and Drug Administration (FDA) [6], growing scientific examination challenges the long-standing perception that NCSs are metabolically inert. Several studies have revealed that their consumption may paradoxically contribute to metabolic dysregulation, glucose intolerance, and even weight gain. NCSs such as aspartame, sucralose, Ace-k, cyclamate, and saccharin are now recognized to interact with multiple physiological systems and be associated with many chronic diseases such as cardiovascular disease and nonalcoholic fatty liver disease, beyond the activation of sweet-taste receptors [7,8]. Notably, evidence has emerged suggesting that chronic consumption of NCSs may disrupt metabolic homeostasis, potentially contributing to insulin resistance and impaired glucose tolerance, effects that paradoxically resemble those associated with excessive sugar intake [9]. A key candidate mediating these effects is the gut microbiota (GM), a diverse ecosystem that plays an integral role in host physiology.

### 1.3. The Gut–Brain Axis as Mediator

Within this context, the gut–brain axis (GBA) represents a complex, bidirectional communication network linking the central nervous system (CNS) with the enteric nervous system (ENS) of the gastrointestinal tract [10]. In particular, multiple signals from the gut microbiota (GM) reach the brain also through the autonomic nervous system and the vagus nerve, as well as via neuroendocrine and immune pathways through the hypothalamic–pituitary–adrenal axis, immune cells, and cytokines mediating inflammation and GBA signaling [10]. The vast and diverse community of microorganisms living in the human intestine takes the name of GM that significantly influences brain health through the production of neurotransmitters (e.g., GABA, serotonin precursors), short-chain fatty acids (SCFAs), and the immune-microbiota loop by which the microbial community modulates innate and adaptive immunity, and immune mediators (e.g., cytokines, pattern recognition receptors) influence microbial composition [11]. Microbial dysbiosis has been linked to impaired appetite regulation and disrupted energy homeostasis, with the GBA playing a key role in mediating these effects [12]. By altering the composition, diversity, and functionality of GM, NCSs can induce dysbiosis and further impair gut–brain communication, potentially contributing to metabolic dysregulation (e.g., glucose intolerance) [13], disordered eating behaviors [14], and altered food reward processing as showed in consumers of NCS-added beverages (i.e., diet soda drinkers) [15]. Despite some conflicting data, studies in both animal models and humans have shown that certain sweeteners (e.g., sucralose) can induce shifts in bacterial taxa associated with metabolic alterations [16,17], as well as for instance for aspartame consumption that was showed to increase fasting glucose levels and the abundance of *Enterobacteriaceae* and *Clostridium leptum* in obese rats [18]. There is therefore evidence that NCSs may alter neural circuits and neurochemical signaling involved in both satiety and reward processing, potentially exacerbating the consumption of ultra-processed, energy-dense foods [19], through mechanisms involving the GBA, the hypothalamus, amygdala, and dopaminergic signaling pathways of the mesocorticolimbic circuit [20,21].

Food intake is a complex and dual process regulated by a dynamic interplay between homeostatic mechanisms, which control energy intake and energy balance, involving the hindbrain and hypothalamic circuitry, and non-homeostatic or hedonic processes orchestrating pleasure-driven eating behavior [22]. Homeostatic feeding is primarily coordinated and integrated by the hypothalamus through different nuclei such as arcuate nucleus (ARC), lateral hypothalamus (LH) and ventromedial hypothalamus (VMH), and hormonal signals such as insulin, leptin, ghrelin, and glucagon-like peptide-1 (GLP-1) mediating body weight homeostasis and the sensing of energy/nutritional status [23].

Not only chronic but simply acute consumption of sucralose was shown to induce an increase in both hunger and hypothalamic blood flow with no variations in peripheral blood glucose levels [21]. Moreover, this study also demonstrated that, during sucralose consumption, there is an increase in functional connectivity between the hypothalamus and brain areas involved in attention and motivational processing such as the anterior cingulate cortex [21].

On the other hand, hedonic feeding is driven by pleasurable food-associated sight, taste, smell, feelings or sensations and is primarily mediated by the mesolimbic reward system, particularly the dopaminergic and oxytocinergic circuits of the ventral tegmental area (VTA), nucleus accumbens (NAc), and prefrontal cortex (PFC), and is influenced by palatability, sensory stimuli, and emotional state [22]. By uncoupling sweetness from caloric content [15,24], NCSs may induce derangements of body weight control and disrupt reward processing [25], leading to increased hedonic drive and intake of palatable foods [25,26]. Thus, NCS-induced dysbiosis has the potential to disrupt the delicate coordination between sweetness perception, caloric intake, and food reward processing.

### 1.4. Aim of the Review

This review explores how NCSs may disrupt the gut–brain axis by inducing microbiota dysbiosis and altering the neural pathways that govern food intake. Section 2 focuses on their effects on the GM and homeostatic feeding circuits, while Section 3 examines how these disruptions extend to dopaminergic reward circuits, altering brain reward pathways and hedonic feeding. By integrating these perspectives, we highlight how artificial sweeteners may not only fail to mimic sugar but also rewire the neurobiological pathways that regulate feeding behavior.

## 2. NCSs, Gut Microbiota and Homeostatic Feeding

### 2.1. Not All Sweeteners Are “Born” Equal

As observed, despite NCSs have been considered metabolically inert due to their low or non-existent caloric content, recent evidence support the view that NCSs can alter GM bacterial ecosystem and metabolic health, appetite and food preference. However, not all NCSs have the same effects on host physiology. Saccharin, sucralose and steviol glycosides appear to support the view. Eight weeks of sucralose consumption in mice has been shown to change GM microbial abundance (i.e., of *Clostridiumcluster*) [27]. A similar outcome was described in rats, where the administration of Splendia^®^ (essentially containing sucralose) for 12 weeks produced the reduction in beneficial microflora (e.g., bifidobacteria and lactobacilli) [28]. In another study in mice, not only sucralose but also Ace-k and saccharin were reported to have a bacteriostatic effect and, in particular, sucralose to change the *Firmicutes*/*Bacteroidetes* ratio [29]. These shifts are frequently associated with impaired glucose tolerance and low-grade inflammation. Remarkably, an increased risk of glucose intolerance in both humans and rodents after prolonged NCS consumption was shown to be mediated by GM alteration [13]. In this study, fecal transplantation from mice consuming saccharin in mice on standard diet was shown to induce impaired glucose tolerance and gut dysbiosis, characterized by the increased abundance of *Bacteroides* and certain *Clostridiales* species, with significantly underrepresented species such as *Akkermansia muciniphila* (*A*. *muciniphila*), in which reduction has been reported in association to unhealthy aging and depression [30,31]. Although with different responses depending on differences in microbiota composition between individuals, NCS consumption in humans was described to be positively correlated with increased adiposity (e.g., weight and waist-to-hip ratio), elevated fasting blood glucose, and impaired glucose tolerance test [13]. However, some recent review studies have contributed to clarify that there is no a systematic correspondence between cross-sectional studies and clinical trials in which NCSs were consumed and alterations in GM composition; some NCSs such as saccharin, sucralose, aspartame, and Stevia are at higher risk to produce changes in GM diversity [32,33,34]. These compositional changes set the stage for functional consequences that directly impact host metabolism and feeding behavior.

### 2.2. Translational Evidence in Humans

Recent clinical and population-based research has begun to substantiate the microbial and metabolic perturbations caused by non-caloric sweeteners (NCSs) that were initially identified in animal studies. A landmark randomized controlled trial, provided some of the most direct human evidence linking NCSs to microbiome-dependent metabolic outcomes. In this 120-participant study, healthy adults were assigned to consume saccharin, sucralose, aspartame, or stevia daily for two weeks. Distinct subsets of individuals, “responders” versus “non-responders”, exhibited variable glycemic responses and microbial compositional shifts, particularly following saccharin and sucralose exposure. Importantly, fecal microbiota transplants from human responders into germ-free mice transferred the impaired glucose tolerance phenotype, confirming that NCS-induced metabolic effects are microbiota-mediated and causally transmissible across species. Beyond controlled interventions, evidence from large-scale cohorts has strengthened the epidemiological link between chronic NCS exposure and metabolic dysfunction. Analyses from the NutriNet-Santé cohort (over 100,000 adults) revealed that higher intake of total and specific NCSs, particularly aspartame, Ace-k, and sucralose, were associated with increased incidence of cardiovascular disease [35], and type 2 diabetes, independent of body mass index, physical activity, and diet quality [36].

Complementary metabolomic studies identified circulating erythritol, a commonly used sugar alcohol, as a biomarker associated with platelet activation and increased risk of major adverse cardiovascular events such as atherothrombotic disease risk [37], suggesting mechanistic vascular consequences of chronic NCS exposure. Together, these converging clinical, cohort, and metabolomic data underscore that NCS-induced alterations in microbial ecology and host metabolism are not restricted to experimental models but extend to human populations. Integrating these findings provides a crucial translational bridge linking gut microbiota disturbances, metabolic dysregulation, and behavioral outcomes along the GBA.

### 2.3. Microbial Metabolites and Neuroactive Compounds

Dysbiosis induced by NCSs may have functional consequences for host physiology through an altered microbial ecosystem. Specifically, NCS-induced changes have been associated with (1) a reduction in the production of SCFAs such as butyrate and propionate [38]; (2) increased intestinal permeability, potentially due to loss of mucin-degrading and tight-junction-stabilizing microbial species [13,39,40]; (3) elevated circulating endotoxin (e.g., lipopolysaccharide, LPS) levels in systemic circulation, often exacerbated by high-fat diet, contributing to low-grade inflammation and insulin resistance [41,42,43].

NCSs may selectively alter specific gut microbial populations that modulate both satiety-promoting metabolites such as SCFAs, serotonin and GLP-1, and appetite-enhancing signals such as GABA [44]. These shifts can disrupt the regulation of homeostatic feeding, potentially leading to overeating and metabolic dysregulation. A recent study in rabbits found that particularly *Bacteroides* species are enriched in GABA-producing pathways [44]. Intragastric GABA administration suppressed satiety hormones (i.e., GLP-1, PYY, CCK) and stimulate feeding behavior. Mechanistically, GM-derived GABA is hypothesized to inhibit satiety hormone secretion, which, through the vagus nerve, disinhibits orexigenic hypothalamic neuropeptide Y (NPY) and the Agouti-related protein (AgRP) neurons (NPY/AgRP), thereby enhancing appetite [44]. Interestingly, one study reported that sucralose intake may promote energy imbalance and increase appetite via an NPY-dependent mechanism [45]. If both saccharin and Ace-k are capable of shifting gut microbiota composition and increasing *Bacteroides* abundance [13,42], it is conceivable that chronic intake of specific NCSs may increase appetite by engaging GABA-mediated inhibition of satiety signals and disinhibition of orexigenic neuropeptides such as NPY. Although direct evidence for NCS-induced GABA alterations remains limited, increased or disrupted *Bacteroides* diversity may enhance GABA production, potentially affecting enteric neurons and appetite control via the GBA. Furthermore, sucralose ingestion in rats has been shown to increase the abundance of *Bacteroides fragilis* (*B. fragilis*) and circulating pro-inflammatory cytokines while reducing occludin expression, a key tight-junction protein [41]. These effects were exacerbated when sucralose was combined with a high-fat diet, leading to metabolic endotoxemia, weight gain, adiposity, and glucose intolerance [41]. Similarly, germ-free mice colonized with microbiota from saccharin-exposed donors exhibited a marked increase in *B. fragilis*, mirroring the donor profile [13]. Since *B. fragilis* has been identified as a GABA-producing bacterium [46,47], its proliferation under NCSs exposure may help explain the observed increases in appetite, body weight, and altered glucose metabolism.

### 2.4. Gut Barrier Function and Inflammation: Diet as a Moderator

Adding to the evidence of a harmful relationship between NCSs and dysbiosis, six weeks of sucralose consumption in mice was shown to induce gut microbiota dysbiosis, notably increasing the relative abundance of *Pseudomonadota* (formerly *Proteobacteria*) and promoting *Escherichia coli* overgrowth [48]. Given the large taxonomic and functional diversity within this phylum, caution is warranted when interpreting such increases, which may reflect overrepresentation of specific taxa rather than a uniform phylum-wide effect.

The metabolic and GBA-associated effects of NCS consumption appear to be influenced by the composition of the consumed diet. For example, the exposure to HFD can amplify the adverse impact of NCSs on microbial diversity, intestinal barrier integrity, and endotoxemia [41], whereas fiber-rich diets may attenuate these effects by promoting microbial resilience and enhancing SCFA production. Similarly, the fecal analysis of rats consuming aspartame for eight weeks revealed an increased abundance of *Enterobacteriaceae* and members of the *Clostridium leptum* group [18]. It should be noted that the genus *Clostridium* encompasses both commensal and pathogenic species. While *C. leptum* and related taxa are generally nonpathogenic and participate in short-chain fatty acid production, other members such as *Clostridioides difficile* and *C. tertium* are classified as risk group 2 pathogens. Throughout this review, references to *Clostridium* or *Clostridiales* are contextualized according to their functional and ecological roles rather than implying uniform pathogenicity.

Interestingly, although the combination of aspartame and HFD attenuated the typical increase in the *Firmicutes*:*Bacteroidetes* ratio observed in models of obesity and type 2 diabetes, it simultaneously promoted a pronounced expansion of *Enterobacteriaceae* species [18]. Notably, such expansion of *Enterobacteriaceae* species in dysbiotic conditions is frequently associated with a reduction in SCFA-producing taxa, resulting in reduced butyrate availability, a pro-inflammatory gut environment, and endotoxemia [49,50]. On the contrary, dietary fiber fosters the growth of beneficial taxa such as *Faecalibacterium prausnitzii* (*F. prausnitzii*) and *A. muciniphila*, both of which are sensitive to NCS-induced reductions and are known to enhance gut barrier function and stimulate satiety hormone release [51,52]. Thus, the impact of NCSs on metabolic health likely depends not only on dose and duration, but also on the overall dietary matrix, particularly the balance between fermentable fibers and pro-inflammatory nutrients like saturated fats. Both prebiotic consumption and adherence to the Mediterranean diet have been shown to increase *F. prausnitzii* and *A. muciniphila* abundance, microbes linked to enhanced satiety and lower energy intake [51,52]. Butyrate, a SCFA produced by *F. prausnitzii* [53], stimulates PYY secretion, promotes satiety, and improves insulin sensitivity [54,55]. Meanwhile, *A. muciniphila* breaks down mucin to generate acetate and propionate, which activate free fatty acid receptors (FFAR2/GPR41 and FFAR3/GPR43) and stimulate GLP-1 and PYY release [56]. Human and animal studies have consistently shown that NCS exposure is associated with reduced *A. muciniphila* abundance. For instance, 11 weeks of saccharin intake lowered its levels in human gut microbiota [13], while maternal Ace-K exposure decreased its presence in newborns [57]. Chronic dysbiosis contributes to low-grade metabolic endotoxemia, driven by elevated LPS levels that promote systemic inflammation, impair gut barrier function, and disrupt vagal signaling related to satiety [58]. Moreover, low-grade metabolic endotoxemia can activate hypothalamic microglia, thereby contributing to defective leptin signaling and the development of leptin resistance [59]. Specific NCSs, including Ace-K and sucralose, alter gut microbial composition and stimulate LPS production, contributing to inflammatory cascades and metabolic dysfunction [41,42]. LPS exposure has also been shown to impair vagal afferent signaling by inducing leptin resistance, thereby reducing CCK-mediated satiety and increasing food intake in rodents [60]. NCS consumption has therefore been associated with alterations in gut microbial composition that have been linked to leptin resistance in preclinical studies; however, causality in humans remains unproven.

### 2.5. Direct Influence of NCSs on Hypothalamic Circuits

If NCSs induce gut dysbiosis (e.g., by promoting the expansion of *Enterobacteriaceae* and reducing SCFA-producing taxa), it should be emphasized that hypothalamic circuits and hormonal factors may also be directly affected by NCS consumption. This dual influence can trigger a “vicious circle” involving both indirect (via the GBA) and direct effects on brain circuits. Notably, NCSs have been associated with an increased risk of leptin resistance [61]. In male mice, chronic consumption of steviol glycosides attenuated activation of the JAK2–STAT5 signaling cascade, the main pathway mediating leptin’s anorexigenic effects [61]. In the same study, sucralose supplementation reduced the expression of anorexigenic POMC-positive neurons in the hypothalamus [61], a critical target of leptin in appetite suppression. As discussed further below (Section 3), NCSs can decouple sweet taste perception from caloric feedback, producing maladaptive effects on brain mechanisms regulating homeostatic feeding. Under physiological conditions, leptin signaling activates anorexigenic neurons in the arcuate nucleus (ARC) [62], while simultaneously inhibiting ARC orexigenic NPY/AgRP neurons [63]. Remarkably, chemogenetic activation of ARC NPY and AgRP neurons increases consumption of sucrose but not of non-caloric saccharin [64]. Consistently, AgRP neurons are inhibited by intragastric food infusion in proportion to the caloric impact of nutrients [65,66], whereas ingestion of a non-caloric but palatable substance (e.g., a sucralose-based calorie-free gel) fails to suppress AgRP neuron activity [66]. If NPY/AgRP neurons are tuned to caloric rather than non-caloric signals, NCSs may fail to inhibit their activity despite sufficient energy availability. This persistent activation of hunger-driving neurons could represent a key mechanism by which prolonged NCS consumption promotes overeating. Another experimental study suggested that the observed increases in food intake and body weight in male rats consuming saccharin for 10 weeks were associated with elevated ghrelin receptor mRNA expression in the hypothalamus [67].

Together, these findings support the idea that chronic NCS consumption can directly affect hypothalamic circuits by reducing leptin-induced POMC activation (blunting satiety), sustaining orexigenic NPY/AgRP activity (driving hunger), and potentially enhancing ghrelin signaling (further promoting food intake). Hence, experimental evidence suggests that certain NCSs may directly influence hypothalamic circuits, although direct effects in humans remain to be confirmed. This convergent disruption of homeostatic feeding pathways may represent a key mechanism linking prolonged NCS use to overeating and weight gain.

### 2.6. Emerging Insights

LPS-mediated systemic inflammation induced by NCSs may disrupt homeostatic regulation of appetite and promote increased energy intake. While a meta-analysis of 20 human randomized controlled trials found no significant changes in serum leptin levels following NCS consumption [68], molecular docking studies indicate that aspartame and sucralose exhibit high binding affinity for the leptin receptor [69]. As emerging mechanisms, this may suggest potential for receptor internalization (i.e., desensitization) or competitive inhibition, further contributing to leptin resistance. *Lactobacillus* and *Bifidobacterium* species, including *L. gasseri*, *L. johnsonii*, *B. longum*, and *B. lactis*, are recognized for their roles in satiety regulation and energy homeostasis due to their capacity to stimulate GLP-1 release and enhance leptin and melanocortin precursor POMC signaling [70,71]. These psychobiotic strains have garnered attention for their ability to modulate GBA activity via synthesis and regulation of neuroactive compounds, notably serotonin (5-HT) [72,73]. Serotonin plays a central role in both mood and metabolic regulation, influencing appetite, energy balance, and even neurodegeneration [31,74]. Notably, *L. acidophilus* and *B. longum* upregulate serotonin transporter (SERT) expression in intestinal epithelial cells [75], thereby increasing serotonergic signaling within the gut. The interplay between peripheral 5-HT production and brain 5-HT is demonstrated by the capacity of the GM to alter systemic tryptophan levels, a precursor of 5-HT, thus affecting its cerebral turnover [72]. For instance, supplementation with *Bifidobacterium infantis* has been shown to elevate plasma tryptophan concentrations [76], facilitating its transport across the blood–brain barrier and subsequently enhancing central 5-HT synthesis [77]. However, NCSs such as sorbitol and sucralose have been linked to declines in beneficial taxa, including *Bifidobacterium*, *Lactobacillus*, and *Bacteroides* [28,78], suggesting that chronic exposure may diminish gut-derived serotonin and impair serotonergic regulation of appetite and energy balance [79,80]. Moreover, species of *Lactobacillus* and *Bifidobacterium* also support host metabolism via SCFA production and immune modulation, which are critical for regulating feeding and body weight. SCFAs stimulate the secretion of GLP-1 and PYY, and metabolites like acetate enhance POMC expression while suppressing AgRP, collectively promoting satiety [81,82]. Given that some NCSs (e.g., sucralose, saccharin, aspartame) reduce SCFA levels, particularly butyrate and propionate [13,38,42], and that these are predominantly produced by *F. prausnitzii* and *Bacteroides* spp., NCS-induced dysbiosis may impair microbial metabolite production, blunting satiety hormone release such as GLP-1 and PYY, and disrupting enteroendocrine signaling and central homeostatic-regulating circuits such as POMC/AgRP signaling.

### 2.7. Limitations, Inconsistencies, and Potential Confounders

Despite increasing evidence that NCSs influence gut microbial composition and metabolic processes, findings across studies remain heterogeneous. Not all sweeteners exert equivalent effects. Sucralose and saccharin have been consistently associated with microbial shifts, while steviol glycosides often yield milder or variable responses. These discrepancies likely reflect differences in chemical structure, metabolic fate, and experimental design, including dosage, duration, host species, sex-dependent responses and background diet. In humans, causal inference remains limited. While some mechanistic insights arise from fecal microbiota transplant studies demonstrating transmissible glucose intolerance or metabolic endotoxemia in mice [13,41], these experiments rely on high-dose exposures that do not necessarily replicate habitual human intake. Indeed, an important translational limitation of current preclinical research lies in the dose disparity between rodent and human exposures. Many rodent studies administer NCS concentrations several-fold higher than the human acceptable daily intake (ADI) established by regulatory agencies, often adjusted per body weight but without accounting for metabolic rate differences. Nevertheless, even sub-ADI doses have been shown to modify microbial composition and glycemic responses in “responder” individuals, indicating possible interindividual sensitivity within human exposure ranges. Sex-dependent effects of NCSs are gaining recognition but remain underexplored. Human data are sparse, yet sex differences in sweet taste preference, energy regulation, and reward sensitivity are well-documented and could modulate NCS effects [83]. Systematic inclusion of both sexes, along with hormonal status or menstrual phase, in future studies will be critical to accurately define NCS impacts on metabolism and feeding behavior. Moreover, epidemiological studies linking NCSs intake to diabetes or cardiovascular risk provide associative data but cannot fully disentangle effects of diet composition, reverse causation (e.g., individuals at metabolic risk preferentially consuming “diet” products), or pre-existing microbiome alterations. Additional confounders may also include dietary matrix or diet composition, so that high-fat vs. fiber-rich diets can modulate NCS effects on microbial diversity and barrier integrity differently. To aid comparison, Table 1 summarizes some key studies focused on the impact of NCSs on GM ecosystem.

### 2.8. Summary

**Figure 1:**
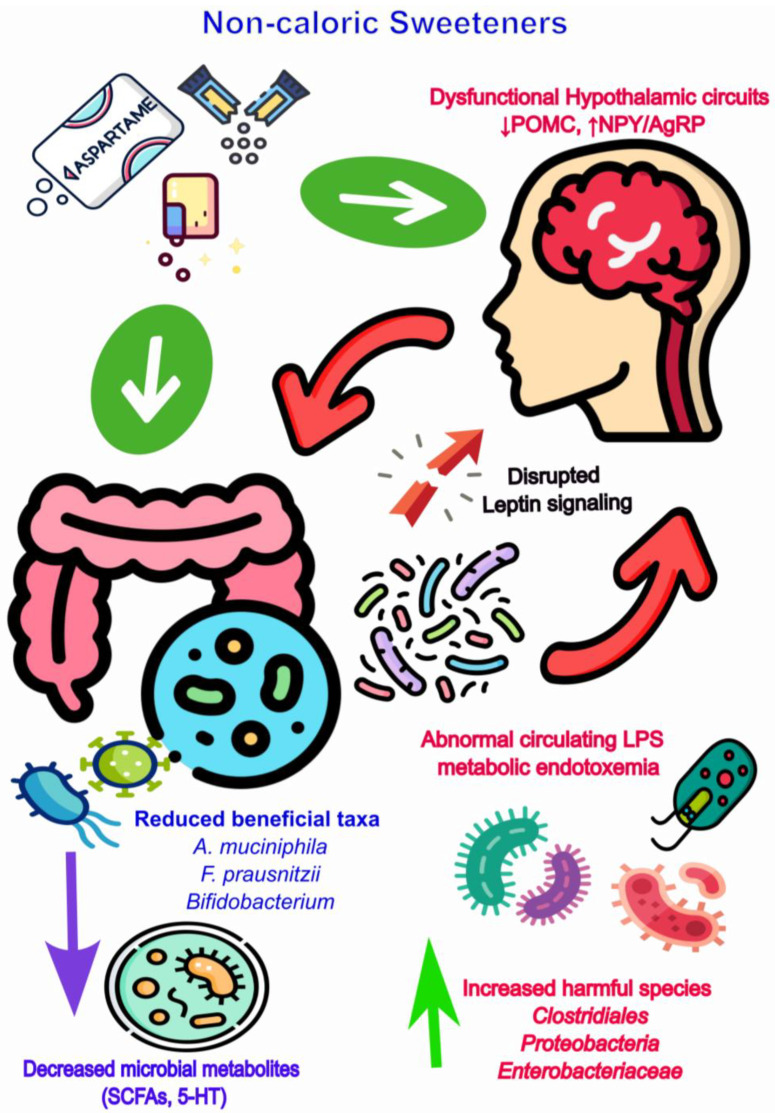
NCS-induced gut dysbiosis reduces SCFA-producing beneficial taxa (e.g., *A. muciniphila*, *F. prausnitzii*, *Bifidobacterium*) and induce proliferation of potentially harmful or opportunistic species within the *Clostridiales* order (e.g., *Clostridium sensu stricto* group, *C. difficile*), *Pseudomonadota* (formerly *Proteobacteria*) and *Enterobacteriaceae* family. These shifts decrease the availability of key microbial metabolites and serotonin-modulating taxa (*Bifidobacterium*, *Lactobacillus*), potentially increasing GABAergic signaling, leading to disruption of gut barrier integrity, and elevated circulating LPS and metabolic endotoxemia. Together, these processes impair satiety hormone release, promote leptin resistance, and alter hypothalamic circuits (↓ POMC, ↑ NPY/AgRP), both indirectly through dysbiosis and directly via disrupted leptin signaling. The resulting imbalance between anorexigenic and orexigenic pathways promotes increased appetite and energy homeostasis dysregulation. Note: It is important to note that the order *Clostridiales* encompasses both beneficial and pathogenic species. While certain members (e.g., *Clostridium butyricum*, *Faecalibacterium prausnitzii*) exert anti-inflammatory and butyrate-producing effects, others (e.g., *Clostridium difficile*) are opportunistic pathogens. In this review, the term *Clostridiales* is used contextually to refer to either beneficial butyrate-producing or potentially harmful taxa, depending on their metabolic and ecological functions.

Collectively, these findings indicates that NCS-induced dysbiosis can impair homeostatic feeding through multiple, interconnected mechanisms. Alterations in gut microbial composition reduce the availability of beneficial metabolites, compromise satiety hormone signaling, and promote systemic inflammation, while some NCSs may directly influence hypothalamic circuits. These processes converge to disrupt appetite regulation and energy balance, with outcomes that are further modulated by dietary context such as fiber intake or high-fat consumption. Clarifying how specific microbial and neuroendocrine pathways interact in response to individual NCSs remains an important goal for mechanistic research. Such insights could inform microbiome-targeted strategies aimed at mitigating the adverse metabolic consequences of NCS use. Figure 1 summarizes these interlinked processes, illustrating how NCS-driven dysbiosis reshapes gut microbial composition, metabolite production, and GBA signaling collectively undermine homeostatic feeding regulation.

## 3. Non-Caloric Sweeteners and Neural Reward Processing

Having outlined how NCS-induced dysbiosis disrupts gut microbial metabolites and homeostatic satiety circuits (Figure 1), we now turn to their effects on hedonic and reward pathways. In this section, we examine how NCSs reshape dopaminergic reinforcement, neuropeptidergic modulation, and gut microbial contributions to reward processing, concluding in an integrated model of food reward disruption.

### 3.1. Evolutionary Foundations of Sweet Taste and Nutritional Coupling

Sweet taste fulfills an essential evolutionary role. Humans evolved a preference for sweetness because it signaled calorie-rich, energy-dense foods such as fruits and honey, with glucose and fructose providing efficient energy sources and glucose serving as a critical fuel for the brain [84,85]. In ancestral environments, where food availability was uncertain, individuals preferring sweet foods gained a survival advantage. Sweetness also produces analgesic effects [84], reinforcing its role as a protective signal. The perception of “sweet” and “umami” tastes is mediated by T1R family receptors, specifically the T1R2/T1R3 heterodimer [86,87]. In evolutionary terms, sweetness reliably predicted caloric density and guided foraging strategies to maximize energy intake. The introduction of NCSs, however, dissociates sweetness from calories. This mismatch creates a form of nutritional decoupling [88], the brain “expects” caloric reward after sweet taste stimulation, but none follows. NCSs can mimic “sweetness” taste by activating T1R2/T1R3 receptors, but they do not deliver calories. This decouples the sensory signal (sweetness) from the nutritional reward (energy). Non-providing usable energy and/or not raising blood glucose levels, NCSs induce a mismatching between the sensory cue (sweet taste) and the post-ingestive caloric outcome (energy intake). In evolutionary terms, caloric sugars aligned sweetness with energy delivery, reinforcing adaptive foraging. In contrast, NCSs activate sweet receptors without post-ingestive caloric feedback, creating a mismatch that may impair weight regulation and food reward processing [89].

It remains unclear how this sensory–nutritional decoupling translates into long-term eating behaviors in humans, as most mechanistic data are derived from animal studies.

### 3.2. NCSs and Dopaminergic Reward Circuits

The dissociation between sweet taste and caloric content can considerably disrupt hedonic feeding. By impairing satiety signaling, this uncoupling may enhance drive for sweet or energy-rich foods, those that provide the strongest expected reward.

Although a full description of hedonic circuits is beyond the scope of this review, several key pathways must be mentioned. Central to this system is the mesocorticolimbic circuit, which integrates signals related to motivation and reward. Within this network, the ventral tegmental area (VTA) projects dopaminergic (DA) outputs to the ventral striatum (nucleus accumbens, NAc), amygdala, and prefrontal cortex (PFC) [90]. While often labeled as part of the “hedonic circuit,” the VTA is more than a pleasure center, it also integrates inputs from vagal and hypothalamic sources, making it a crucial hub for energy-related information and motivational cues associated with eating (e.g., food-seeking) and food reward within the mesocorticolimbic circuit. Food palatability and DA signaling are tightly coupled and mechanistically intertwined. Palatable foods reliably elevate extracellular DA levels in the NAc [91]. Reward-related behaviors typically unfold in two stages: (i) an appetitive phase (food seeking or pursuit of reward), and (ii) a consummatory phase (reward delivery), where the mesolimbic pathway encodes the hedonic value of food [92,93].

Highly palatable foods can prolong consumption, leading to overeating and metabolic dysregulation. Supporting this, optogenetic studies show that stimulating VTA DA neurons during feeding prolongs intake, while inhibition reduces it [94]. Interestingly, administration of GLP-1 receptor agonists such as semaglutide reduces VTA DA activity and suppresses palatable food consumption. However, direct activation of VTA DA neurons can override this suppression, highlighting the powerful role of dopaminergic signaling in opposing satiety signals [94]. “Real” sugars, such as sucrose, engage both sweet taste receptors (T1R2/T1R3) and post-ingestive caloric feedback. This dual activation strongly reinforces sensory input and robustly stimulates VTA DA neurons, producing a positive feedback loop between taste and calories. In contrast, NCSs activate taste receptors but fail to provide post-ingestive energy signals. This mismatch may disrupt DA-dependent reward prediction error signaling, weakening the expected reinforcement of sweetness. While it is established that sugar consumption engages both taste and nutrient-sensing systems, it remains uncertain whether these systems converge on overlapping or distinct neural pathways when comparing caloric vs. non-caloric sweeteners (e.g., sucrose vs. sucralose).

In a seminal study, Tellez et al. [95] illustrated this dissociation. Using a model where mice received sucralose paired with intra-gastric delivery of either glucose or sucralose, the authors found distinct DA responses in striatal subregions. DA release increased in the ventral striatum (VS) after both sucralose and glucose infusion, but elevations in the dorsal striatum (DS) occurred only with glucose. Moreover, when sucralose’s palatability was reduced with a bitter additive (while still delivering glucose), DA release in the vs. disappeared, while DS activation persisted [95]. These findings suggest that the vs. encodes hedonic aspects of taste, while the DS encodes nutritional properties of sugar.

This distinction has important implications for NCSs. Because they engage taste receptors without caloric reinforcement, NCSs are likely to produce weaker or absent DA responses in the VTA and related circuits, thus emphasizing the importance of caloric content, not just taste, in reward processing. Such blunted signaling may trigger compensatory food-seeking or overeating in an attempt to “restore” reward prediction. Supporting this, studies show that NCS consumption weakens conditioned satiety responses. For instance, rats exposed to saccharin pressed levers more frequently for sucrose pellets than controls, suggesting increased food-seeking behavior driven by the absence of expected post-ingestive reward [96]. Unlike sucrose, which engages both sweet taste receptors and caloric reinforcement to robustly activate DA pathways, NCSs stimulate taste receptors alone, producing weaker or absent dopaminergic responses and potentially driving compensatory food seeking [96]. It is still unresolved whether dopaminergic encoding of caloric vs. non-caloric sweeteners converges on overlapping circuits or recruits distinct pathways in humans.

### 3.3. Beyond Dopamine: Neuropeptidergic Modulation of Sweetness and Reward

While DA is central to reward processing, other neuromodulators, including orexin, neurotensin (NTS), and oxytocin, also play critical roles in linking sweetness to hedonic value. These systems interact with dopaminergic circuits, often amplifying or modulating responses to sweet taste. The hypothalamus is a key regulator of this integration. It connects homeostatic energy regulation with hedonic pathways via strong anatomical and functional links from hindbrain, midbrain, limbic, hypothalamic, and cortical regions to the VTA [97]. Imaging studies in humans illustrate these dynamics: ingestion of glucose produces a rapid and robust hypothalamic response, while fructose and sucrose generate weaker, slower activations [83]. In contrast, sucralose produces only a transient hypothalamic signal, but a sustained VTA response similar to water. However, sugars such as glucose, fructose, and sucrose resulted in reduced VTA activation compared to water. These findings suggest that NCSs fail to reproduce the coordinated hypothalamic-VTA activation characteristic of caloric sugars, reflecting the decoupling of taste and metabolic signals (e.g., caloric content) [98]. A similar study compared in adult individuals the effects of different types of sugars such as glucose, fructose, allulose or sucralose on satiety signaling extrapolated as BOLD activation in a distributed neural network including cingulate cortex, brainstem, VTA, insula, hypothalamus and basal ganglia [99]. The intake of a glucose-sweetened shake resulted in diminished BOLD responses in key basal regions of the brain, including the cingulate cortex, brainstem, VTA, and insula, and glucose was the only sugar that produced an increase in connectivity in the “salience network” identified in the anterior cingulate cortex (that is important for executive control) and orbital frontoinsular cortices [99,100]. On the contrary, the BOLD signal remained unchanged following the ingestion of sucralose and allulose, indicating that both sweeteners did not influence brain areas related to feeding behavior. This further suggests that sweetness alone is insufficient to evoke the brain activity changes commonly linked to both satiety and the “salience network” [99]. NCSs also induce molecular and cellular adaptations. For example, saccharin consumption increases DA and noradrenaline turnover in the striatum while decrease both in the PFC [101]. Moreover, while both sucrose and saccharin decreased neuronal activity (c-Fos expression) in the orbitofrontal cortex (OFC), saccharin but not sucrose consumption induced an increase in c-Fos expression in the anterior insular cortex and motor cortex [101]. These brain regions are involved in risk-taking and decision-making strategies, and this study also evaluated the degree to which saccharin and sucralose can reduce the ability to select flexible choices, showing behavioral rigidity during the decision-making process [101]. The increased activity in the anterior insular cortex may thus explain the impairment in flexibility about reward-based decisions, shifting behavior toward rigidity and weakening hedonic valuation within the OFC–NAc circuit.

Mechanistically, such saccharin-induced alterations are linked to an imbalance in DA D2 receptor (D2R) expression, upregulated in the striatum but downregulated in the PFC [86,87]. Interestingly, prolonged early-life exposure to Ace-K has also been shown to reduce tyrosine hydroxylase (TH) expression in the VTA, thereby diminishing DA biosynthesis and increasing later-life sugar (i.e., fructose) intake [102]. Collectively, these findings suggest that NCSs can rewire reward pathways, particularly during sensitive neurodevelopmental periods, causing neuroadaptations that affect reward sensitivity, and seeking behavior.

The lateral hypothalamus (LH) is another important hub, integrating homeostatic and hedonic signals. This region contains glutamatergic and GABAergic neurons, as well as orexin, melanin-concentrating hormone (MCH), and neurotensin (NTS) neurons [103,104]. Inputs from ARC NPY/AgRP and POMC neurons regulate hunger and satiety [103,105], while projections from the amygdala, bed nucleus of the stria terminalis (BNST), and NAc convey reward and stress-related signals [106,107,108]. Moreover, amygdala and PCF send glutamatergic projections to the LH [109]. Efferent LH pathways, including orexinergic and NTS projections, innervate the VTA and NAc, amplifying reward sensitivity and promoting food seeking [110,111,112,113]. The LH also provides inputs from orexin- and MCH-positive neurons to the NAc [114], as well as GABAergic and orexinergic inputs to the PFC and amygdala [115]. Thus, the orexinergic projections from the LH to the VTA and NAc are strongly activated by cues associated with food rewards, and convey signals to enhance reward sensitivity, palatability signals and reward seeking [116,117]. Notably, orexin release into the ventral pallidum has been identified as part of a “hedonic hotspot” that intensifies sucrose pleasure responses [104]. Pharmacological blockade of orexin receptors reduces saccharin self-administration, highlighting orexin’s critical role in hedonic responses to sweetness, independent of caloric content [118]. Consistently, saccharin exposure has been shown to increase c-Fos expression in orexin-expressing neurons within the LH, without parallel changes in MCH neurons [119]. Importantly, both orexin and MCH neurons in the LH are modulated by NTS-expressing neurons, which project to the VTA and promote DA release and reward-related behaviors [120,121].

It is recognized that prolonged consumption of HFD can blunt preference toward hedonic food, decreasing the hedonic value of palatable and caloric food [90]. NTS also modulates hedonic feeding. A recent study has provided mechanistic evidence that chronic HFD consumption reduce NTS expression and trigger a decrease in NTS signaling in NAc-to-VTA projecting neurons, thus blunting VTA DA activation and lowering the hedonic value of food [122]. Although direct effects of NCSs on NTS remain unproven, their indirect impact on orexin and dopaminergic circuits suggests convergence on similar mechanisms [104,105,107]. Because NTS regulates DA signaling within the NAc [122], and sucralose intake has been linked to elevated ΔFosB expression in this region [123], NCSs may drive molecular adaptations resembling those observed in addictive states.

As previously noted, saccharin consumption decreases neuronal activity in the OFC while concurrently elevating c-Fos expression in the anterior insular cortex [101]. Functionally, the OFC plays a central role in assigning value to food-related stimuli, as fMRI studies demonstrate its involvement in encoding the anticipated outcomes of food rewards [124]. In the context of reward prediction network, which includes the VTA and its mesocortical projections, the OFC helps evaluate discrepancies between expected and actual rewards [125]. Within this circuit, oxytocin adds another regulatory layer. Acting through receptors in the VTA and NAc, oxytocin dampens DA responses to food cues and decreases intake of palatable foods [126]. Both rodent and human studies show that exogenous oxytocin administration suppresses sugar seeking, even in the absence of hunger [127]. Conversely, loss of oxytocin leads to excessive consumption of sweeteners such as sucrose and saccharin, emphasizing its role in constraining hedonic feeding [128].

Taken together, these findings illustrate that NCSs influence reward not only by weakening dopaminergic prediction error signaling, but also by reshaping neuropeptidergic modulation. Dysregulation of orexin, NTS, and oxytocin signaling, particularly within VTA–NAc–PFC pathways, may contribute to maladaptive food seeking, impaired reward sensitivity, and greater susceptibility to overeating. These neuroadaptive changes indicate a broader disruption in the integration of sensory, hormonal, and reward-related inputs, processes known to be directly shaped by GBA interactions. NCSs not only blunt dopaminergic reinforcement but also reshape neuropeptidergic signaling (orexin, NTS, and oxytocin), altering decision-making flexibility and hedonic valuation within VTA–NAc–PFC networks [128].

However, direct causal links between specific NCSs and long-term neuropeptidergic adaptations remain poorly defined, particularly in humans where evidence is limited to imaging correlates.

### 3.4. Gut Microbial Mediators of NCS-Induced Alterations in Hedonic Feeding

As previously detailed, chronic consumption of NCSs such as saccharin, sucralose, and Ace-K can drastically alter gut microbial diversity, promote the proliferation of pro-inflammatory taxa, and disrupt microbial-derived signaling molecules implicated in homeostatic regulation. However, these microbiota-induced shifts may also contribute to dysregulation of hedonic feeding, by influencing neural and peptidergic signals along the mesocorticolimbic pathways. By causing reductions in SCFA-producing bacteria (e.g., *Lactobacillus*, *Faecalibacterium*, *Roseburia*), NCS consumption can also have a detrimental impact on hedonic or non-homeostatic feeding. Germ-free (GF) mice showed increased DA and 5-HT turnover in the striatum [129], and depletion of the GM by antibiotic treatment in mice produced a robust increase in high-palatable sucrose food consumption and enhanced motivation for sucrose-food reward [130] as well as higher consumption of high-fat high-sucrose diet [131].

We previously described that consumption of saccharin and Ace-K can suppress satiety signals and induce glucose tolerance, effects that were accompanied by an abnormal rise in *Bacteroides* abundance [13,30,31,42]. Administration of *Bacteroides uniformis* through fecal microbiota transplantation has been reported to enhance dopamine transporter (DAT) binding within the striatum [120], which in turn can diminish extracellular DA availability and blunt dopaminergic signaling via a reduced activation of postsynaptic DA receptor. DAT overexpressing transgenic mice showing increased DAT function and about ~40% decrease in extracellular DA concentrations, show also reduced motivation to work to obtain food reward and sensitivity to natural reward [132]. Similar results were obtained with mice overexpressing striatal DA D2 receptors that exhibited reduced motivation (i.e., incentive motivation) necessary to obtain a reward, akin to anhedonia-like behavior [133]. The DA synthesized by GM may influence mesolimbic DA activity through a variety of indirect mechanisms. GM can modulate the availability of DA precursors, particularly L-3,4-dihydroxyphenylalanine (L-DOPA), which can cross the BBB and be converted via decarboxylation to DA in the brain. There is evidence that some microbial species are capable to affect L-DOPA metabolism, and in particular that *Enterococcus faecalis*-producing tyrosine decarboxylases enables L-DOPA metabolism, increasing its bioavailability and DA synthesis [134].

The ability of selected probiotics (e.g., *Lactobacillus acidophilus*, *Lactobacillus casei*, *Lactobacillus plantarum*, and *Bifidobacterium longum*) to produce beneficial effects on mental health, depressive symptoms, dementia and autism spectrum disorder is increasingly documented [31,135]. The administration of *Lactobacillus casei* in a rat model of depression-like behavior has been shown to produce an increase in DA levels in the frontal cortex [136]. Both *Enterococcus faecalis* and *Enterococcus faecium* can modulate DA levels, as showed in a neuroimaging study in which their transplantation in a mouse model of Parkinson’s disease (PD) was shown to increase the amount of striatal DA [137]. Sucralose consumption in rats has also been associated with the suppression of beneficial bacteria such as lactobacilli while minor effects on detrimental bacteria such as enterobacteria were found [28]. Consumption of sucralose for ten weeks in healthy volunteers was shown to reduce the abundance of *Lactobacillus acidophilus* [138]. Thus, consumption of specific NCSs can negatively interact with GM ecosystem and induce detrimental changes in bacterial diversity and abundance of microbial species, and affect mesolimbic DA signaling. There are several studies corroborating the view that probiotics can exert a protective action against neurotoxins targeting DA signaling, as demonstrated in animal models of PD in which the administration of probiotic mixture including *Lactobacillus rhamnosus* GG, *Bifidobacterium animalis lactis*, and *Lactobacillus acidophilus* counteracted the decrease in DA levels and protected from DA neurons loss [139]. Similarly the administration of *Lactobacillus plantarum* DP189 exerted neuroprotection in a MPTP model of PD, in particular by inhibiting the apoptosis of dopaminergic neurons and increasing the number of TH-positive cells [140]. In another study, the oral administration of a cocktail of different *Lactobacillus* such as *Lactobacillus fermentum* LH01, *Lactobacillus reuteri* LH03, and *Lactobacillus plantarum* LH05 demonstrated protection of both the gut barrier and the BBB and prevention of the dopaminergic neuronal loss induced by striatal 6-OHDA lesion [141].

*A. muciniphila* is a gut bacterium that has received a great deal of attention for its anti-inflammatory and intestinal-barrier-protection properties as well as for its potential beneficial in multiple diseases such as diabetes, obesity, cancer, and metabolic syndrome [142]. A similar attention has also received the multifaced role of *A. muciniphila* in neurological and psychiatric conditions [31,143], including the possible functional link between physical exercise, irisin production and BDNF expression in the brain [30]. The indirect modulation exerted by *A. muciniphila* upon brain DA content has received experimental confirmation by an elegant study reporting the reduction in this bacterium in the feces of rats that underwent to the 6-OHDA model of PD, and the subsequent improvement of motor deficits and protection from DA neuronal loss due to *A. muciniphila*-dependent increase in butyrate production [144]. Saccharin and sucralose consumption has been shown to be responsible for drastic changes in GM composition, including in particular an important depletion in the abundance of *A. muciniphila* together with disruption of intestinal permeability and systemic inflammation [145]. Exposure during pregnancy to Ace-K (plus sucralose) was shown to induce dysbiosis in the offspring, which resulted characterized by a marked depletion of *A. muciniphila* colonization [57].

The genus *Clostridium* encompasses an extensive and taxonomically diverse lineage within the phylum *Bacillota*, including both commensal and pathogenic representatives. Clusters such as IV (the non-pathogenic *Clostridium leptum* group, comprising *Faecalibacterium prausnitzii*) are major producers of butyrate and are associated with intestinal and neuronal health [146]. In contrast, other species such as *Clostridioides difficile* and *Clostridium tertium* are opportunistic pathogens belonging to risk group 2 for humans and animals. For clarity, in this review, the term “*Clostridium*” refers to specific beneficial, SCFA-producing taxa implicated in gut–brain axis homeostasis, rather than the entire genus. Studies indicate that NCS exposure (e.g., sucralose) can markedly reduce the abundance of these *Clostridiales* clusters [27], potentially disrupting butyrate-mediated protection of dopaminergic neurons [147,148]. Sodium butyrate possesses the ability to protect dopaminergic neurons from death via multiple mechanisms such as inhibition of α-synuclein [148], further supporting the evidence that butyrate-producing bacteria (e.g., *F. prausnitzii*, *Roseburia* and *Eubacterium*) can exert selective neuroprotective action against DA neuronal degeneration.

It is suggestive therefore to hypothesize that, by depleting some gut *Clostridium* species and *A. muciniphila* colonization, the consumption of NCSs such as saccharin, sucralose and Ace-K can lead to reduced butyrate production together with reduced neuroprotection of DA neurons against different environmental threats and pathogenetic factors. Besides butyrate, there is evidence that microbial metabolites such as indole derivatives and propionate can modulate NAc activity and reduce the palatability of high-energy foods in humans, thus illustrating how microbiota influence reward circuits. In nonobese healthy individuals, it was reported that an increase in colonic production of propionate reduced the anticipatory reward responses elicited by the evaluation of high- or low-energy dense food pictures [149]. In particular, by using the fMRI paradigm, the authors established a reduction in BOLD signal in caudate and NAc associated with a decrease in responses elicited by pictures of high-energy foods, thus demonstrating that an increase in propionate production in the GM can contribute to reduce reactivity to food and to the control reward-driven eating behavior. *Bacteroides* such as *B. thetaiotaomicron* have been described as capable of producing propionate [150]. While no direct evidence currently links NCS consumption to altered colonization of *B. thetaiotaomicron*, artificial sweeteners have been shown to interfere with carbohydrate metabolism. *B. thetaiotaomicron* is particularly noted for its ability to degrade dietary polysaccharides (starches, fibers, host glycans) through specialized outer membrane systems known as Polysaccharide Utilization Loci (PULs) [151,152]. Some NCSs (e.g., sucralose, saccharin, Stevia derivatives) inhibit α-amylase or α-glucosidase activity in vitro, enzymes structurally and functionally related to bacterial PUL enzymes [153]. This suggests that NCSs could compete with natural polysaccharide substrates for enzyme binding sites, thereby impairing starch and fiber degradation. A reduction in polysaccharide metabolism would not only lower the colonization efficiency and energy yield of *B. thetaiotaomicron* but also decrease SCFA production, including propionate. This represents another potential pathway by which the consumption of NCSs could reduce SCFA production and alter DA signaling, thereby promoting the intake of highly rewarding foods. Feeding pregnant rats with a combination of high fat diet and aspartame or Stevia was shown to increase adiposity in the offspring, alter GM ecosystem and the expression of genes of the mesolimbic system involved in the consumption of rewarding palatable food [154]. Nine-weeks’ consumption of the Stevia component Rebaudioside A was reported to alter GM composition and decrease tyrosine hydroxylase (TH) and DA transporter (DAT) mRNA expression in the NAc [155]. Moreover, three-weeks of intermittent access to Stevia intake produced an increase in the transcription factor ΔFosB immunoreactivity in NAc and caudate putamen, supporting the idea that Stevia consumption may increase DA activity in brain areas playing a key role in reward and addictive behaviors [156]. Interestingly, the hedonic drive to consume not only drug of abuse or sucrose but also Stevia, along with ΔFosB-positive cells, was reduced in animals reared in conditions of environmental enrichment [156] as also observed for the reduction in rewarding effects mediated by drug of abuse such as cocaine [157].

If modulation of the GM through prebiotic supplementation can reduce food-related hedonic and motivational drives, then the combination of prebiotics with NCSs such as Stevia could help normalize DA markers (e.g., TH, DAT) and mesolimbic-driven hedonic control of feeding, thereby reducing the consumption of palatable, rewarding foods. Notably, supplementation with fructo-oligosaccharides (FOS) has been shown to counteract alterations in hedonic feeding and the reinforcing effects induced by a high-fat/high-sugar diet [158]. This study further support the idea that FOS may favor the expression of genes involved in the NAc DA signaling leading to a reduction in palatable food intake.

Caloric sugars promote SCFA-producing bacteria (e.g., *Lactobacillus*, *Clostridium*, *A. muciniphila*) that support DA signaling and neuroprotection, whereas NCSs deplete these taxa, impair microbial metabolite production (SCFAs, propionate, L-DOPA), and exacerbate DA dysregulation [116,117,118,119,120,121,122,123,124,125,126,127,128,131,132,133,134,135,136].

Although compelling animal evidence links NCS-induced dysbiosis to altered reward processing, direct human studies connecting microbial shifts with brain DA signaling remain scarce. The evidence reviewed highlights converging top-down and bottom-up mechanisms through which NCSs reshape food reward processing.

### 3.5. Integrative Perspective

Taken together, NCSs disrupt food reward pathways through two converging mechanisms: (1) direct effects on neural circuits; caloric sugars engage both T1R2/T1R3 receptors and post-ingestive caloric signals, producing robust dopaminergic reinforcement and coordinated hypothalamic-VTA activation [90]. Thus, NCSs have been reported to modulate food reward pathways in animal models, but the relevance and consistency of these effects in humans are not yet established. In contrast, NCSs provide sensory sweetness without caloric confirmation, weakening dopaminergic prediction error signaling; disrupting OFC-mediated value coding; and altering neuropeptidergic modulators such as orexin, NTS, and oxytocin [90]. These changes impair hedonic valuation, promote inflexibility in reward-based decisions, and may amplify compensatory food seeking. (2) indirect effects via the GM; caloric sugars support microbial ecosystems that produce SCFAs (butyrate, propionate) and L-DOPA, metabolites known to modulate striatal DA signaling and protect dopaminergic neurons [122,134,135,136,137]. NCSs, however, deplete beneficial taxa (e.g., *A. muciniphila*, *Clostridium*, *Lactobacillus*) [125,132], impair SCFA production, and may interfere with microbial carbohydrate metabolism (e.g., through inhibition of PUL enzymes) [138,139,140]. These microbial disruptions reduce DA availability and neuroprotection, contributing to maladaptive hedonic feeding.

By dissociating sweetness from calories, NCSs introduce a dual disruption of the GBA: top-down (neural circuits failing to receive caloric confirmation of sweetness) and bottom-up (microbial ecosystems failing to supply neuromodulatory metabolites). These parallel pathways converge on altered dopaminergic turnover, ΔFosB accumulation, receptor imbalances, and impaired neuropeptidergic regulation. Importantly, while caloric sugars promote reinforcement and energy homeostasis (at a metabolic cost), NCSs appear to foster reward instability and compensatory seeking behaviors. However, despite robust animal evidence, human translational studies are still scarce. Key gaps include whether (i) NCS-induced dysbiosis directly alters DA signaling in humans; (ii) how early-life NCS exposure affects neurodevelopmental wiring of reward circuits [87], and (iii) whether prebiotics or targeted probiotics (e.g., SCFA-producing strains) can counteract NCS-induced disruptions [145]. To aid comparison, Table 2 summarizes the principal mechanisms involved, opposing the reinforcing actions of caloric sugars with the disruptive effects of NCSs, alongside key supporting references.

**Figure 2:**
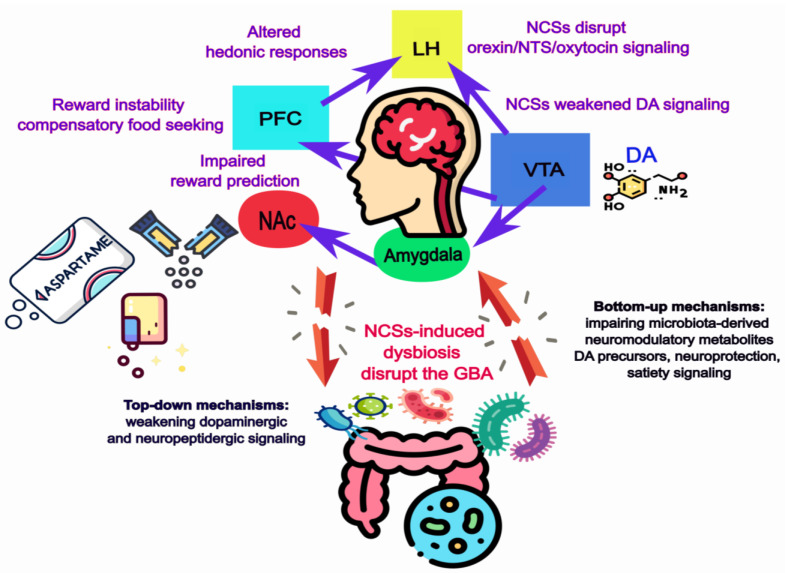
Mechanistic pathways by which NCSs disrupt food reward processing. (**A**) Dopaminergic reward circuits. Caloric sugars activate both sweet taste receptors and post-ingestive caloric signals, producing robust dopaminergic reinforcement in the VTA, NAc, and PFC. In contrast, NCSs activate taste receptors without caloric confirmation, leading to weakened DA signaling, impaired reward prediction, and compensatory food seeking. (**B**) Neuropeptidergic modulation. Hypothalamic peptides (orexin, NTS, oxytocin) amplify or constrain hedonic responses via projections to the VTA and NAc. Caloric sugars preserve balanced modulation, while NCSs, contributing to rigid reward-based decisions and altered hedonic valuation. (**C**) Gut microbial contributions. Sugars support SCFA production and beneficial taxa within the *Clostridiales* order (e.g., *Clostridium butyricum*, *Faecalibacterium prausnitzii*, *Roseburia*), along with *A. muciniphila* and *Lactobacillus*. These *Clostridium*-related taxa belong to nonpathogenic, butyrate-producing clusters that promote dopaminergic precursors, neuroprotection, and satiety signaling, distinct from pathogenic *Clostridioides* species. NCSs induce dysbiosis (loss of SCFA producers, enrichment of pro-inflammatory taxa), impairing DA turnover, barrier integrity, and microbial metabolite signaling. (**D**) Integrated perspective. NCSs disrupt the GBA through two converging pathways: (i) top-down, weakening dopaminergic and neuropeptidergic circuits; and (ii) bottom-up, impairing microbiota-derived neuromodulatory metabolites. Together, these changes foster maladaptive hedonic feeding, reward instability, and compensatory seeking, in contrast to the coordinated reinforcement and metabolic balance supported by caloric sugars. Blue arrows mean dopaminergic connections.

Next, a schematic overview integrating neural and microbial contributions is shown in Figure 2.

Taken together, the evidence reviewed in Section 3 highlights how NCSs disrupt hedonic feeding through parallel neural and microbial mechanisms (see Figure 2). By weakening dopaminergic and neuropeptidergic circuits while simultaneously impairing microbiota-derived support for reward signaling, NCSs emerge as dual disruptors of the gut–brain axis.

## 4. Conclusions and Outlook

Non-caloric sweeteners represent a double-edged innovation, providing sweetness without calories yet disrupting the biological coherence between sensory pleasure and metabolic feedback. By weakening dopaminergic reinforcement and deranging microbial ecosystems, NCSs may foster a state of “reward instability”, maladaptive hedonic feeding, and compensatory seeking behaviors that promotes overeating and metabolic dysregulation. Despite compelling mechanistic insights, several limitations persist. Most evidence derives from animal studies, inter-individual variability in GM composition remains underexplored, and long-term human data are limited. Future directions should focus on integrative clinical studies combining microbiota profiling, neuroimaging, and behavioral outcomes to clarify how NCSs reshape food reward systems. Moreover, forthcoming research should pursue longitudinal, multi-omics approaches integrating microbiome, metabolome, and connectome analyses. Clinical trials evaluating prebiotic and probiotic interventions could determine whether microbiota restoration restores healthy reward processing. In the author’s view, redefining beneficial sweetness through the lens of the gut–brain axis offers an opportunity to reconcile pleasure and sustainability in nutrition and healthy aging.

## Figures and Tables

**Table 1:** Summary of representative studies investigating the impact of NCSs on GM composition and metabolic outcomes in animal models and human studies. Studies vary widely in species, exposure dose, and duration.

Study (Ref)	Model/Design	Sweetener (s)	Exposure Duration/Dose	Key Microbiota Changes	Main Metabolic Findings
**Suez et al., 2014 [13]**	Mouse (C57BL/6J); Fecal microbiota transfer	Saccharin, Sucralose and Aspartame in follow-up human validation	Mice: 11 weeks of saccharin in drinking water (0.1 mg/mL, comparable to ADI); Humans: 7-day exposure to saccharin at FDA-acceptable daily intake levels	In mice: ↑ *Bacteroides*, ↑ certain *Clostridiales* species; ↓ *Lactobacillus reuteri* and *Akkermansia muciniphila* induce dysbiosis. Fecal microbiota transplantation from saccharin-fed mice transferred glucose intolerance to germ-free recipients.	Glucose intolerance and insulin resistance in both mice and subset of human participants. Demonstrated causality between NCS-induced dysbiosis and host metabolic impairment.
**Bian et al., 2017 [42]**	CD-1 mice	Ace-K	**Duration:** 4 weeks; **Dose:** 37.5 mg/kg body weight/day (equivalent to human acceptable daily intake) via drinking water	Sex-dependent alteration of gut microbial diversity and composition: ↓ *Lactobacillus* and *Clostridium* (females); ↑ *Bacteroides* and Sutterella (males); enrichment of genes involved in energy metabolism and xenobiotic degradation.	Sex-dependent effects: **males** showed ↑ body weight, altered lipid metabolism; **females** showed no body weight gain. Ace-K modulates metabolic outcomes through microbiota-dependent mechanisms.
**Uebanso et al., 2017 [27]**	C57BL/6J mice;	Ace-K, Sucralose	**Duration:** 8 weeks; **Dose:** low-dose sucralose (1.5 mg/kg) or high-dose sucralose (15 mg/kg). Ace-K (15 mg/kg)	Sucralose ↓ *Clostridium* cluster XIVa. Sucralose specifically reduced total SCFA concentrations (notably butyrate)	No changes in body weight or fasting glucose, changes in SCFA profiles and bacterial taxa suggest early dysbiosis. Even low-dose, chronic NCS exposure can modify gut ecology.
**Palmnäs et al., 2014 [18]**	**male Sprague–Dawley rats** on **high-fat diet (HFD)** or **standard chow**	Aspartame (low dose; 5–7 mg/kg/day via drinking water)	8–12 weeks (chronic exposure during diet feeding)	↑ *Enterobacteriaceae*, ↑ *Clostridium leptum*, altered overall bacterial diversity; increased fecal propionate (SCFA) levels	No major weight gain difference; ↑ fasting glucose, ↓ insulin sensitivity, altered glucose tolerance; metabolic effects linked to microbiota-derived propionate
**Olivier-Van Stichelen et al., 2019 [57]**	Pregnant and lactating C57BL/6J mice; progeny monitored post-weaning	Sucralose (0.1 mg) and Ace-K (0.25 mg) as upper limit ADI; Sucralose (0.2 mg) and Ace-K (0.5 mg) as twice ADI	Gestation + lactation (maternal exposure only; offspring unexposed after weaning)	Offspring microbiota altered ↓ *A*. *muciniphila*; overall increase in Firmicutes	Metabolic deregulation in offspring, in particular glycine metabolism (potential decrease in glutathione synthesis)
**Abou-Donia et al., 2008 [28]**	Sprague-Dawley Male Rat	Splenda^®^ (sucralose-based) by oral gavage at 100, 300, 500, or 1000 mg/kg	12 weeks	↓ *Bifidobacteria*, ↓ *Lactobacilli*, ↓ *Bacteroides*,	decrease in beneficial intestinal bacteria, histopathological changes in the colon (e.g., lymphocytic infiltrates into epithelium, increased body weight)
**Rodriguez-Palacios et al., 2018 [48]**	Mice	Splenda^®^ (sucralose-based) “low dose” (1.08 mg/mL); “high dose” (3.5 mg/mL) to drinking water	6 weeks	↑ *Proteobacteria*, ↑ *E. coli*	Gut dysbiosis, endotoxemia
**Debras et al., 2022 [35]**	Human prospective cohort (NutriNet-Santé)	Mixed NCSs (Aspartame, Ace-K, Sucralose)	Median follow-up 9 years	N/A (epidemiological)	Aspartame ↑ Cerebrovascular disease risk; Ace-K, Sucralose ↑ coronary heart disease risk
**Debras et al., 2023 [36]**	Human cohort (NutriNet-Santé)	Mixed NCSs (Aspartame, Ace-K, Sucralose)	Median follow-up 9.1 years	N/A	↑ Type 2 diabetes incidence

Note: Throughout this review, bacterial taxa are described according to current nomenclature. The phylum *Proteobacteria* is referred to as *Pseudomonadota*, and the genus *Clostridium* is recognized as taxonomically heterogeneous, including both commensal and pathogenic species. References to *Clostridium* here pertain specifically to commensal, SCFA-producing taxa unless otherwise stated. ↑ increase and ↓ decrease.

**Table 2:** Summary of mechanistic pathways by which NCSs alter food reward processing.

Mechanism	Caloric Sugars (Effects)	NCSs (Effects)	Key References
Dopaminergic reward circuits	Activate sweet receptors + post-ingestive caloric signals → robust DA release in VTA, NAc, PFC; strong reward prediction	Activate receptors without calories → weakened DA signaling, impaired prediction error, compensatory food seeking	[74,75,79,80,81,82]
Neuropeptidergic modulation	Balanced orexin, NTS, oxytocin signaling supports hedonic control and decision flexibility	Dysregulated orexin/NTS/oxytocin → rigid reward-based choices, altered hedonic valuation	[85,103,104,105,106,107,108,109,110,111,112,113,114,115]
Microbial contributions	Support SCFA producers (*A. muciniphila*, *Clostridium*, *Lactobacillus*); enhance DA precursors, neuroprotection, satiety	Dysbiosis: loss of SCFA-producers, enrichment of pro-inflammatory taxa; impaired DA turnover, barrier integrity	[13,27,28,29,30,31,32,33,34,35,36,37,38,116,117,118,119,120,121,122,123,124,125,126,127,128,131,132,133,134,135,136]
Integrated perspective	Coordinated top-down (neural) and bottom-up (microbiota) reinforcement → stable reward + energy homeostasis	Dual disruption: weakened neural reinforcement + impaired microbial metabolite support → reward instability, overeating	[87,138,139,140,141,142,143,144,145]

## Data Availability

No new data were created or analyzed in this study. Data sharing is not applicable to this article.

## Abbreviations

The following abbreviations are used in this manuscript:

Ace-K	Acesulfame potassium
AgRP	Agouti-related peptide
ARC	Arcuate nucleus
BBB	Blood–brain barrier
BNST	Bed nucleus of the stria terminalis
BOLD	Blood-oxygen-level-dependent
CCK	Cholecystokinin
CNS	Central nervous system
DA	Dopamine/dopaminergic
DAT	Dopamine transporter
DS	Dorsal striatum
ENS	Enteric nervous system
FFAR2/GPR41	Free fatty acid receptor 2
FFAR3/GPR43	Free fatty acid receptor 3
fMRI	Functional magnetic resonance imaging
FOS	Fructo-oligosaccharides
GABA	Gamma-aminobutyric acid
GBA	Gut–brain axis
GF	Germ-free
GLP-1	Glucagon-like peptide-1
GM	Gut microbiota
GRAS	Generally Recognized as Safe
HFD	High-fat diet
LH	Lateral hypothalamus
LPS	Lipopolysaccharide
L-DOPA	L-3,4-dihydroxyphenylalanine
MCH	Melanin-concentrating hormone
NAc	Nucleus accumbens
NCSs	Non-caloric sweeteners/Non-nutritive sweeteners
NPY	Neuropeptide Y
NTS	Neurotensin
OFC	Orbitofrontal cortex
PFC	Prefrontal cortex
POMC	Pro-opiomelanocortin
PULs	Polysaccharide utilization loci
PYY	Peptide YY
SCFAs	Short-chain fatty acids
SERT	Serotonin transporter
TH	Tyrosine hydroxylase
VTA	Ventral tegmental area
VS	Ventral striatum
VMH	Ventromedial hypothalamus
WD	Westernized diet
